# Phenotyping of difficult asthma using longitudinal physiological and biomarker measurements reveals significant differences in stability between clusters

**DOI:** 10.1186/s12890-016-0232-2

**Published:** 2016-05-10

**Authors:** T. Zaihra, C. J. Walsh, S. Ahmed, C. Fugère, Q. A. Hamid, R. Olivenstein, J. G. Martin, A. Benedetti

**Affiliations:** Montreal Chest Institute, McGill University Health Centre, Montreal, QC Canada; Meakins Christie Laboratories, and McGill University, Montreal, QC Canada; Department of Mathematics, The College at Brockport, State University of New York, Brockport, NY USA; Division of Clinical Epidemiology, McGill University Health Centre, Montreal, QC Canada; School of Physical & Occupational Therapy, McGill University, Montreal, QC Canada; Department of Epidemiology, Biostatistics and Occupational Health, McGill University, 1020 Pine Ave. W., Montreal, QC H3A 1A2 Canada; Department of Medicine, McGill University, Montreal, QC Canada; Respiratory Epidemiology and Clinical Research Unit, McGill University Health Centre, Montreal, QC Canada; Keenan and Li Ka Shing Knowledge Institute of Saint Michael’s Hospital, Toronto, ON Canada; Institute of Medical Sciences and Department of Medicine, University of Toronto, Toronto, ON Canada

**Keywords:** Asthma, Phenotype, Longitudinal, Predictors, Exacerbation and Clusters

## Abstract

**Background:**

Although the heterogeneous nature of asthma has prompted asthma phenotyping with physiological or biomarker data, these studies have been mostly cross-sectional. Longitudinal studies that assess the stability of phenotypes based on a combination of physiological, clinical and biomarker data are currently lacking.

Our objective was to assess the longitudinal stability of clusters derived from repeated measures of airway and physiological data over a 1-year period in moderate and severe asthmatics.

**Methods:**

A total of 125 subjects, 48 with moderate asthma (MA) and 77 with severe asthma (SA) were evaluated every 3 months and monthly, respectively, over a 1-year period. At each 3-month time point, subjects were grouped into 4 asthma clusters (A, B, C, D) based on a combination of clinical (duration of asthma), physiological (FEV_1_ and BMI) and biomarker (sputum eosinophil count) variables, using k-means clustering.

**Results:**

Majority of subjects in clusters A and C had severe asthma (93 % of subjects in cluster A and 79.5 % of subjects in cluster C at baseline). Overall, a total of 59 subjects (47 %) had stable cluster membership, remaining in clusters with the same subjects at each evaluation time. Cluster A was the least stable (21 % stability) and cluster B was the most stable cluster (71 % stability). Cluster stability was not influenced by changes in the dosage of inhaled corticosteroids.

**Conclusion:**

Asthma phenotyping based on clinical, physiologic and biomarker data identified clusters with significant differences in longitudinal stability over a 1-year period. This finding indicates that the majority of patients within stable clusters can be phenotyped with reasonable accuracy after a single measurement of lung function and sputum eosinophilia, while patients in unstable clusters will require more frequent evaluation of these variables to be properly characterized.

## Background

Global prevalence, morbidity, mortality, and economic burden associated with asthma have increased over the last 40 years [1]. Despite improved treatments for asthma, updated recommendations for asthma care such as the Global Initiative for Asthma Guidelines (GINA) [2], and various national clinical practice guidelines, lack of asthma control still remains a significant issue [3]. Approximately 300 million people worldwide currently have asthma, and its prevalence increases by 50 % every decade [1]. In North America, 10 % of the population has asthma [1] and the prevalence of asthma in the United States is increasing [4]. Heterogeneity of clinical presentation and disease mechanisms exist within asthma, frustrating attempts to achieve optimal asthma control in all subjects. Identification of asthma phenotypes, i.e. groups with unique characteristics that are stable or predictable over time, may have prognostic or therapeutic significance leading to better tailoring of subject-centered therapies.

Traditional classification and management approaches in asthma do not reflect the heterogeneous characteristics of this disease. Our study contains subjects who meet the American Thoracic Society definition of severe asthma, which supports clinical heterogeneity in asthma and the need for new approaches for the classification of disease severity in asthma. Use of clustering is quite popular in identifying clinical phenotypes of asthma [5–8].

Recent studies have identified different asthma phenotypes based on clinical features and pathophysiological mechanisms [5–8] using cluster analysis. However, the stability of clusters over time has not been well addressed [9]. Moore et al. [5], proposed asthma phenotypes based on physiological variables and medical history, while, Hastie et al. [10] identified phenotypes using biomarkers of inflammation neutrophil and eosinophil count in induced sputum. Kupczyk et al. [11], found that 30 and 49 % of severe asthmatics changed cluster allocation after a 1-year follow up using physiological and biomarker clustering, respectively. The authors concluded that physiological and biomarker measurements reflect different activities of asthma. Therefore we hypothesized that integration of physiological, clinical and biomarker data may provide a more comprehensive profile of each asthmatic and improve the stability of cluster allocation. We analyzed clusters at five time points (at 3-month intervals) over a 1-year span to more accurately assess stability of these clusters.

The purpose of this work was to 1) identify clusters/phenotypes using a combination of clinical, physiological, and biomarkers measurements (i.e. duration of asthma, FEV_1_, BMI, and sputum inflammatory cells), which have each been applied in studies of asthma phenotyping, [5, 6, 8, 10], but have not been used in combination previously; 2) to quantify the degree of change in allocation of these asthma clusters over time (i.e. stability) using longitudinal measurements collected at five time points over a 1-year period in a well-characterized cohort of moderate and severe asthmatics.

## Methods

We analyzed longitudinal data from the difficult asthma cohort of the Montreal Chest Institute of the McGill University Health Centre, comprising 48 moderate and 77 severe asthmatic subjects. Moderate asthmatics were evaluated every 3 months and severe asthmatics monthly over a 1-year period.

### Participants

Subjects aged 18–80 years with moderate or severe asthma were enrolled over a 13-year period in respiratory clinics (the Montreal Chest Institute of McGill University Health Centre and Sacré-Coeur Hospital). Asthma was defined according to the American Thoracic Society (ATS) criteria [12–14]. Subjects were considered as having severe asthma if, on enrolment, they fulfilled 1 major criterion and 2 minor criteria as previously defined by the ATS workshop on difficult-to-treat asthma [15]. Subjects were considered to have moderate asthma if they had asthma controlled on 200–1000 mcg/d fluticasone or equivalent with or without concomitant therapy with a long-acting β-agonist, leukotriene modifier or theophylline; they had no more than 2 steroid bursts in the past year, and none in the past 3 months, with less than 30 total days on oral steroids in the previous year; and they had a forced expiratory volume in 1 s (FEV_1_) > 70 % of the predicted value and > 90 % of personal best from the past 2 years; they had a maximum of one unscheduled visit for asthma in the previous year.

Subjects who had smoked more than 10 pack-years, were current smokers or were known to have other pulmonary diseases, including chronic obstructive pulmonary disease were excluded. The Research Ethics Board of the McGill University Health Centre and the Comité d’éthique de la recherche of Hôpital du Sacré-Cœur de Montréal approved the study. All subjects provided signed informed consent. The research complied with the declaration of Helsinki.

### Measurements/procedures

Most clinical visits were in the mornings. Subjects were not instructed to hold their bronchodilators. We present pre-BD measurements. Symptom severity was graded according to the Juniper asthma control questionnaire (ACQ) [16]. Well-controlled asthma was defined by ACQ < 1.0 [17]. Spirometry was performed according to ATS standards. Allergy skin prick tests with commercial extracts from common allergens were performed using the modified skin prick method, and a test was regarded as positive if the wheal was >3 mm. Subjects were regarded as atopic if they had 1 or more positive allergy tests. Sputum was induced using inhalation of increasing concentrations (3, 4, and 5 %) of hypertonic saline and processed as previously described by Lemière et al. [18]. Exhaled nitric oxide analysis was performed according to ATS guidelines [19]. The nurse study coordinator collected data on changes in treatment monthly.

Subjects were classified as persistent-, intermittent- or non-eosinophilic [20], using longitudinal measures of sputum eosinophil counts. Persistent eosinophilia was defined as eosinophils > 2 % on each sputum analysis, intermittent eosinophilia was defined as at least one occurrence of eosinophils > 2 % on sputum analysis, and non-eosinophilic was defined as eosinophils < 2 % on each sputum analysis. Subjects with less than 3 sputum samples available for analysis were not classified.

### Statistical methods

The data from these subjects have been used for other studies (Walsh CJ, Zaihra T, Benedetti A, Fugère C, Olivenstein R, Lemiere C, Hamid Q, Martin JG. Exacerbation risk in severe asthma is stratified by inflammatory phenotype using longitudinal measures of sputum eosinophils. Submitted to Clinical and Experimental Allergy, 2016.). We took all subjects’ data measurements at baseline, 3, 6, 9, and 12 months for both severe and moderate asthmatics (without averaging the values in between these time point for the severe subjects who had additional visits). For some subjects information was missing on clustering variables: age-of-onset was missing for 4 subjects, FEV_1_ and sputum eosinophil count were missing for 26 and 31 subjects, respectively, at one or more time points. Missing values were imputed using the Amelia package in R that uses a bootstrapping-based algorithm [21, 22]. We used k-means cluster analysis [23] to identify four distinct clusters. We used caution in using the term “phenotype” interchangeably with cluster assignment. While the clustering approach is a tool for identifying groups with similarities in the included variables, it is a step away from defining a phenotype to which an individual patient may be assigned prospectively [24].

We decided on four *a priori* clusters, based on other phenotypic analyses of asthma data and the number of subjects in our dataset [5, 6]. Clustering of the asthma participants in our study was based on a combination of physiological variables and biomarkers (BMI, FEV_1_ (%), years of asthma (YOA), and eosinophil count) at the following five time points: baseline, 3, 6, 9 and 12 months. We calculated pairwise similarity indices for the clusters at different time-points via the Sorensen similarity index [25] which measures similarity between two clusters A and B as SI = 2ab/(a + b), where *a* is the number of subjects found in cluster A; *b* is the number of subjects in cluster B and *ab* is the number of subjects shared by both the clusters. We calculated the similarity index between clusters identified at different time points and then classified clusters that were the most similar as the same cluster over time, i.e. we assessed whether subjects who were grouped together at baseline remained together at each subsequent evaluation. We used a t-test to assess if variables used for clustering changed in subjects who changed clusters from baseline to 12 months.

## Results

One hundred twenty five subjects had a total of 593 clinician assessments at baseline, 3, 6, 9, and 12 months resulting in 593 spirometric tests, 538 FENO measurements, and 400 sputum analyses.

### Baseline clinical characteristics of the asthma participants

Subject characteristics (*n* = 125) are shown in Table 1. There were no significant differences in age, age-of-onset of asthma, sex, smoking history, or atopic status between moderate and severe asthmatics. Prevalence of atopy was high in both groups. Pre-bronchodilator FEV_1_ (%) was significantly lower in severe vs. moderate asthmatics, as was FEV_1_/FVC. Severe asthmatics had significantly higher ACQ score, inhaled corticosteroid (ICS) dosage and percent sputum eosinophils compared to moderate asthmatics.

**Table 1:** Baseline characteristics of the subjects. Values presented are mean (SD) or number of subjects and proportion of subjects [n (%)]

Variable	Severe Asthmatics (*n* = 77)	Moderate Asthmatics (*n* = 48)	*p*-value
Sputum Neutrophils (%)	49.0 (31.2)	43.1 (26.3)	0.32
Sputum Eosinophils (%)	11.8 (17.6)	4.6 (6.5)	0.01
Age	49.9 (12.6)	46.6 (11.2)	0.13
BMI	28.1 (5.8)	27.7 (6.9)	0.74
Age-of-Onset (AOO)	22.9 (18.0)	22.1 (14.7)	0.77
Beclamethasone or equivalent dose (mcg)	1340.7 (524.1)	677.2 (425.5)	<0.01
F_E_NO (ppb)	30.5 (28.6)	20.4 (18.2)	0.02
ACQ	2.0 (1.0)	1.0 (0.8)	<0.01
FEV_1_(%)	63.4 (19.7)	87.2 (12.5)	<0.01
FEV_1_/FVC	64.9 (12.2)	74.1 (8.5)	<0.01
Prednisone user [n (%)]	41 (53 %)	2 (4 %)	<0.01
Omalizumab user [n (%)]	8 (10 %)	0 (0 %)	0.02
Males [n (%)]	43 (56 %)	23 (48 %)	0.50
Non Smoker [n (%)]	52 (67.5 %)	25 (53 %)	0.16
Atopic [n (%)]	58 (81 %)	42 (87.5 %)	0.45

*Abbreviations*: *FEV*_*1*_ Forced Expiratory Volume at 1 s, *ICS* inhaled corticosteroid, *AOO* age of onset of asthma, *ACQ* Asthma Control Questionnaire score, *BMI* body mass index, *F*_*E*_ *NO* fractional of exhaled nitric oxide in parts per billion (ppb)

### Characterization of the baseline clusters

Clusters were formed using baseline data, as well as using the data obtained at the 3, 6, 9 and 12-month evaluations. Tables 2 and 3, present the characteristics of the 4 clusters identified at the baseline and 12-month evaluation. The following descriptions are based on the clusters established from data gathered at the baseline.

**Table 2:** Baseline Cluster Descriptive. Values shown are number of subjects, and percentage of total subjects [n (%)] unless stated

Variable	Phenotype A (*n* = 15)	Phenotype B (*n* = 17)	Phenotype C (*n* = 39)	Phenotype D (*n* = 54)	*p*-value
FEV_1_ < 80 %	12 (80.0)	9 (52.9)	37 (94.9)	20 (37.0)	<0.01
Beclamethasone or equivalent dose >1600mcg	7 (46.7)	2 (11.8)	6 (15.4)	4 (7.4)	<0.01
AOO <20 year	3 (20.0)	7 (41.2)	31 (79.5)	17 (31.5)	<0.01
ACQ ≤ 1	2 (13.3)	5 (29.4)	14 (35.9)	24 (44.4)	0.15
Female	6 (40.0)	10 (58.8)	12 (30.8)	31 (57.4)	0.05
Severe asthmatic	14 (93.3)	10 (58.8)	31 (79.5)	22 (40.7)	<0.01
Atopic	11 (73.3)	13 (76.5)	34 (87.2)	42 (77.8)	0.58
Non-Smoker	9 (60.0)	13 (76.5)	28 (71.8)	27 (50.0)	0.09
Age [mean(SD)]	46.7 (13.4)	44.9 (8.8)	52.4 (12.6)	47.5 (11.9)	0.10
BMI [mean (SD)]	26.2 (3.8)	40.1 (4.4)	27.0 (3.9)	25.4 (3.7)	<0.01
Sputum Neutrophils (%) [mean (SD)]	34.3 (27.4)	51.0 (24.8)	43.6 (29.2)	47.5 (29.3)	0.34
Sputum Eosinophils (%) [mean (SD)]	3.9 (5.3)	33.5 (16.7)	5.1 (6.5)	5.8 (6.0)	<0.01
F_E_NO ppb [mean (SD)]	28.7 (26.5)	25.5 (23.8)	29.7 (30.6)	24.0 (21.4)	0.74

*Abbreviations*: *FEV*_*1*_ Forced Expiratory Volume at 1 s, *ICS* inhaled corticosteroid, *AOO* age of onset of asthma, *ACQ* Asthma Control Questionnaire score, *BMI* body mass index, *F*_*E*_ *NO* fractional of exhaled nitric oxide in parts per billion (ppb). Chi-square test was used for categorical variables, and one-way ANOVA was performed for continuous variables

**Table 3:** Description of Cluster at 12 months. Values shown number of subjects, and percentage of total subjects [n (%)] unless otherwise stated

Variable	Phenotype A (*n* = 25)	Phenotype B (*n* = 16)	Phenotype C (*n* = 33)	Phenotype D (*n* = 51)	*p*-value
FEV_1_ < 80 %	22 (88.0)	8 (50.0)	29 (87.9)	19 (37.3)	<0.01
Beclamethasone or equivalent dose >1600mcg	4 (16.0)	2 (12.5)	5 (15.2)	8 (15.7)	0.99
AOO < 20 year	7 (28.0)	7 (43.8)	30 (90.9)	14 (27.5)	<0.01
ACQ ≤ 1	6 (24.0)	5 (31.2)	13 (39.4)	21 (41.2)	0.48
Female	9 (36.0)	9 (56.2)	11 (33.3)	30 (58.8)	0.07
Severe asthmatic	21 (84.0)	9 (56.2)	24 (72.7)	23 (45.1)	<0.01
Atopic	19 (76.0)	13 (81.2)	27 (81.8)	41 (80.4)	0.95
Non-Smoker	19 (76.0)	13 (81.2)	22 (66.7)	23 (45.1)	0.01
Age [mean (SD)]	49.0 (11.6)	44.4 (8.8)	52.4 (12.4)	47.2 (12.7)	0.12
BMI [mean (SD)]	26.8 (4.4)	40.1 (4.6)	26.5 (4.1)	25.7 (3.7)	<0.01
Sputum Neutrophils (%) [mean (SD)]	45.9 (29.1)	50.0 (25.2)	44.5 (29.2)	43.8 (29.5)	0.90
Sputum Eosinophils (%) [mean (SD)]	6.2 (6.2)	8.5 (6.3)	8.8 (9.9)	31.5 (12.7)	<0.01
F_E_NO (ppb) [mean (SD)]	33.8 (35.1)	23.1 (22.2)	26.6 (23.3)	24.1 (21.5)	0.42

*Abbreviations*: *FEV*_*1*_ Forced Expiratory Volume at 1 s, *ICS* inhaled corticosteroid, *AOO* age of onset of asthma, *ACQ* Asthma Control Questionnaire score, *BMI* body mass index, *F*_*E*_ *NO* fractional of exhaled nitric oxide in parts per billion (ppb). Chi-square test was used for categorical variables, and one-way ANOVA was performed for continuous variables

#### Cluster A

Cluster A comprised 12 % of the subjects (*n* = 15). Most were severe asthmatics (93 %) and predominantly late-onset disease (80 %). The majority of subjects (80 %) in this cluster had poor baseline pre-bronchodilator lung function (FEV_1_ < 80 % predicted). Most subjects (87 %) within this cluster had poor self-perceived asthma control (ACQ ≥ 1) and almost half (47 %) were on very high dose inhaled corticosteroids (ICS > 1600 mcg).

#### Cluster B

Cluster B comprised 14 % of all subjects (*n* = 17). Over half of these subjects (59 %) were severe asthmatics and female (59 %). This cluster was characterized by a higher BMI than the other clusters (mean BMI = 40.1, vs. median 26.2 in other clusters, *p*-value < 0.01). Seventy one percent of subjects had poor self-perceived asthma control (ACQ ≥ 1).

#### Cluster C

Cluster C contained 31 % of the subjects (*n* = 39), most of whom were severe asthmatics (79 %) with reductions in pulmonary function (FEV_1_) at baseline (95 %). Subjects were mainly early onset (80 %), atopic (87 %). This cluster had the greatest proportion of subjects with early asthma onset (79.5 % versus median 31.5 % in other clusters *p* < 0.01). Thirty six percent of subjects in this group perceived their asthma as uncontrolled.

#### Cluster D

Cluster D comprised 54 subjects (43 %). Over half of subjects (60 %) were moderate asthmatics and the majority (63 %) had good lung function (FEV_1_ > 80 %).

### Temporal stability of identified clusters

**Fig. 1:**
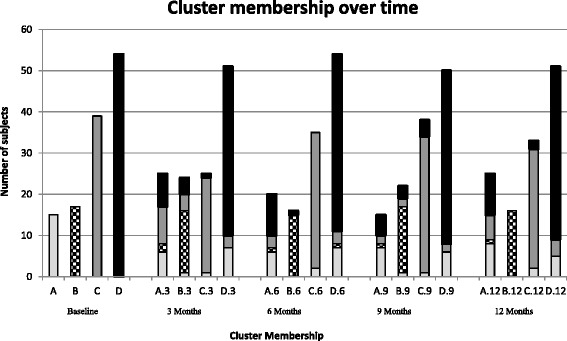
Cluster membership over time^1^, by baseline cluster membership. ^1^Cluster membership at baseline is indicated by the bar colours. The graph depicts how subjects are clustered together over time

Overall, the prevalence range of the clusters across all five-time points was: Cluster A [12–20 %], Cluster B [13–30 %], Cluster C [20–31 %] and Cluster D [40–43 %]. To study temporal stability of the clusters, we estimated the subject flux from one cluster to another along with similarity indices between one cluster and another at baseline, 3, 6, 9 and 12 months (Table 4). Cluster A was the least stable of the 4 clusters; 3 out of 15 subjects (20 %) allocated at baseline to cluster A remained in the same cluster over time. Cluster B was the most stable: 12 out of 17 (71 %) allocated at baseline to cluster B remained together at each time point. Cluster C and D were intermediate: with 20 out of 39 (51 %) clustered at baseline in cluster C staying in the same cluster at each time point, and 31 of 54 (57 %) subjects in cluster D remaining in the same cluster at each time point. Figure 1 displays cluster membership at baseline and how subjects clustered at baseline were clustered at 3, 6, 9 and 12 months.

**Table 4:** Similarity^a^ of baseline clusters to the 3, 6, 9 & 12 months clusters

Baseline Cluster (n)	3 months Cluster Similarity (n)		6 months Cluster Similarity (n)		9 months Cluster Similarity (n)		12 months Cluster Similarity (n)	
A (15)	A	0.30 (6)	A	0.39 (6)	A	0.38 (7)	A	0.40 (8)
A	B	0.05 (1)	B	0.00 (0)	B	0.05 (1)	B	0.00 (0)
A	C	0.05 (1)	C	0.08 (2)	C	0.04 (1)	C	0.08 (2)
A	D	0.35 (7)	D	0.20 (7)	D	0.19 (6)	D	0.15 (5)
B (17)	A	0.10 (2)	A	0.05 (1)	A	0.06 (1)	A	0.05 (1)
B	B	0.73 (15)	B	0.91 (15)	B	0.82 (16)	B	0.97 (16)
B	C	0.00 (0)	C	0.00 (0)	C	0.00 (0)	C	0.00 (0)
B	D	0.00 (0)	D	0.03 (1)	D	0.00 (0)	D	0.00 (0)
C (39)	A	0.28 (9)	A	0.10 (3)	A	0.07 (2)	A	0.19 (6)
C	B	0.13 (4)	B	0.00 (0)	B	0.07 (2)	B	0.00 (0)
C	C	0.72 (23)	C	0.89 (33)	C	0.86 (33)	C	0.81 (29)
C	D	0.07 (3)	D	0.07 (3)	D	0.04 (2)	D	0.09 (4)
D (54)	A	0.20 (8)	A	0.27 (10)	A	0.14 (5)	A	0.25 (10)
D	B	0.10 (4)	B	0.03 (1)	B	0.08 (3)	B	0.00 (0)
D	C	0.03 (1)	C	0.00 (0)	C	0.09 (4)	C	0.05 (2)
D	D	0.78 (41)	D	0.80 (43)	D	0.81 (42)	D	0.80 (42)

^a^Similarity indices between two clusters were calculated using Sorensen’s Index, as SI = 2ab/(a + b), where a is the number of subjects found in cluster A; b is the number of subjects in cluster B and ab is the number of subjects shared by both the cluster

Overall, 53 % (*n* = 66) subjects had stable cluster membership, remaining in clusters with the same subjects at each evaluation time. The remaining changed their clusters, based on either the improvement or deterioration of their condition/risk factors. Specifically, 10 out of 54 subjects from cluster D moved to cluster A over the 12 months follow-up period indicating a shift to a cluster with worse lung function.

At all evaluation times, subjects who changed clusters had a statistically significantly larger average absolute change in sputum eosinophils and FEV_1_ than subjects who did not change clusters (Table 5). Subjects who changed clusters had a higher change in absolute dose of inhaled corticosteroids than subjects who remained in the same cluster, but this was statistically significant only from baseline to 6 months (data not shown).

**Table 5:** Comparison of Mean Absolute Change in Sputum eosinophils (%) and FEV_1_ (%) between subjects who changed cluster as opposed to those who did not change cluster

Flux Period	Patients with no change in cluster	Patients with change in cluster	*p*-value
Baseline to 3 months N	85	40	
Absolute change in FEV_1_(%) Mean (SD)	6.2 (5.6)	12.1 (15.1)	0.02
Absolute change in Eos (%) Mean (SD)	5.9 (6.2)	16.7 (15.1)	<0.01
Baseline to 6 months N	97	28	
Absolute change in FEV_1_(%) Mean (SD)	5.6 (6.0)	16.1 (17.0)	<0.01
Absolute change in Eos(%) Mean (SD)	6.1 (7.3)	22.1 (15.9)	<0.01
Baseline to 9 months N	98	27	
Absolute change in FEV_1_(%) Mean (SD)	6.6 (5.9)	16.7 (16.0)	<0.01
Absolute change in Eos Mean (SD)	7.9 (8.3)	19.9 (14.3)	<0.01
Baseline to 12 months N	95	30	
Absolute change in FEV_1_(%) Mean (SD)	8.3 (7.8)	15.1 (15.1)	0.03
Absolute change in Eos Mean (SD)	7.8 (8.7)	16.3 (14.2)	<0.01

### Similarity indices between time points for each cluster

Similarity indices for cluster A ranged from 0.30–0.40, and for cluster B the range was from 0.73–0.97. The similarity index for cluster C ranged from 0.72–0.89 while for cluster D the range was from 0.78–0.81. Basically, clusters B, C, D were reproducible but cluster A was not.

## Discussion

To our knowledge, this is the first study to demonstrate significant differences in the stability of cluster groupings based on longitudinal physiological and biomarker data. One of the strengths of our study is the novel application of clustering and longitudinal models (“longitudinal clustering”) to characterize asthma phenotypes over a 1-year period. Given the size of dataset we had to pick the clustering variables parsimoniously. We integrated a combination of clinical, physiological, and biomarker variables selected based on the results from multiple previous clustering papers. For example, Moore et al. [5] found that FEV1(%) and age were the major determinants of phenotype. Sutherland et al. [26] found obesity (BMI) to be an important determinant of asthma in adults. Sputum % eosinophils were selected because these have been found to be an important biomarker indicating exacerbation risk [6]. We avoided including % Neutrophils because of an inherent inverse correlation between %Neutrophils and % Eosinophils (most notable at the extreme values). Our goal was therefore to assess stability of clusters using a combination of these rather well established “markers” of phenotype.

We identified 4 clusters at baseline characterized on age of onset, FEV_1_, BMI, and sputum eosinophilia from 125 well-characterized adults with moderate to severe asthma. We then evaluated phenotypic stability using 5 longitudinal observations at 3-month intervals over a 1-year period. Comparing each of the clusters at each of the five time points we found that each cluster remained qualitatively similar indicating reproducibility of the clustering method at each time point to classify data into groups having similar characteristics. We observed that some asthmatic subjects displayed marked variability in lung function and/or sputum eosinophils and this resulted in significant differences in degree of stability between the clusters. This finding indicates that some clusters may allow reasonable (i.e. roughly 70 %) specificity of phenotype classification after obtaining clinical data and just one measurement of lung function and sputum eosinophils, while other clusters will require multiple measurements for proper characterization.

We sought to determine whether each cluster may have an overrepresentation of other clinical characteristics that would suggest a common pathophysiologic mechanism. Cluster B, the most stable cluster, contained a high proportion of obese women who tended to be less symptomatic and used less inhaled corticosteroid than subjects allocated to other clusters. While not all subjects in this cluster were non-eosinophilic, the eosinophil counts were lowest in this group. There are potentially several reasons why obese subjects may be more symptomatic for a given level of disease activity. Results from bariatric surgery suggest that the impairment relates to the mechanics of breathing, in particular breathing at low lung volumes [27]. Lack of awareness of obesity as a predominantly mechanical constraint is likely to lead to over-treatment for presumed airway inflammation.

Previous studies that have used clustering to define phenotypes of asthma have used differing types of data for their clustering and the majority have only used cross-sectional data. Haldar et al. [6] identified 4 distinct clinical phenotypes in a population of refractory asthma subjects, including a cluster composed of predominantly obese females with significant asthma symptoms. This phenotype cluster corresponds to our cluster B asthmatic subjects; Halder et al found that inhaled corticosteroid doses could be reduced in these subjects without worsening their asthma control. The Severe Asthma Research Program (SARP) of the US National Heart, Lung and Blood Institute characterized five distinct clinical phenotypes of asthma based on a cluster analysis of 726 asthma subjects [5]. They identified three common variables namely; baseline forced expiratory volume in 1 s (FEV1), maximal FEV1 after albuterol and age at asthma onset and used them in functional tree analysis. Among the primary care cohort, some of the characteristics that stood out among the more severe clusters were female, atopy, high BMI, and sinusitis [7]. These studies, and ours, indicate that there are some consistent markers independent of the population or the clustering techniques used. These include gender (prominence among females), atopy, blood eosinophil count, age of onset and fixed airflow limitation. Kim et al. [8] described four phenotypes from two Korean cohorts on more than 2500 severe asthmatics. Their phenotypes were discriminated by FEV_1_, age of onset and smoking.

Few papers have addressed the temporal stability of phenotypes. Kim et al. [8] addressed transitions from one phenotype to another, observed 10 years apart and concluded that a subject’s phenotype showed a some consistency over time, with probability of membership in the same asthma phenotype at both times ranging from 54 to 88 %*.* They also observed different transition patterns across phenotypes, with transitions towards increased asthma symptoms more frequently among non-allergic phenotypes as compared to allergic phenotypes. The relative lack of sensitivity of non-Th2 associated asthma to inhaled corticosteroid therapy could account for this latter phenomenon [28]. Kupczyk et al. [11], concluded that phenotypes determined by biomarkers are less stable than those defined by physiological variables, especially in severe asthmatics. In contrast, the most volatile phenotype in our study was composed mainly of subjects with poor baseline pre-bronchodilator lung function, poorly self-perceived asthma control and who were often on very high dose inhaled corticosteroids. Such subjects also had sputum eosinophils and FEV_1_ values that tended to fluctuate the most – resulting in unstable clusters. Moreover, changing FEV_1_ and eosinophil values may prompt changes in ICS doses.

Our study has a number of limitations. The sample size is relatively small and there were some missing data. We used imputation for missing data. Furthermore, unsupervised statistical learning techniques (e.g. k-means cluster analysis) sometimes lead to inconsistent results due to variable selection and demographic and clinical differences among study populations. Prosperi et al., [24] concluded that observed heterogeneity within clusters may reflect real differences in demographic and clinical characteristics but may also be an artefact of the clustering techniques and the choice of variables analyzed. However, we carefully selected variables that may be important in defining asthma phenotypes rather than the product of asthma.

## Conclusions

Our results suggest that focusing on identifying clusters of patients by measuring patients at any one given time-point may be unreliable. Perhaps the use of endotypes will permit more meaningful characterization [29] and true personalization of asthma management. Future work will consider using information from more than one time point to identify phenotypes.

Future studies are needed to evaluate the use of phenotypes prospectively to screen individuals at risk for adverse future outcomes and to improve asthma control by personalizing asthma management. The significant role of subject-reported outcomes such as ACQ indicates a strong need for primary care services to ensure optimal management of asthma, to prevent exacerbations and to reduce the long-term burden of this disease.

## Ethics approval and consent to participate

The Research Ethics Board of the McGill University Health Centre and the Comité d’éthique de la recherche of Hôpital du Sacré-Cœur de Montréal approved the study. All subjects provided signed informed consent. The research complied with the declaration of Helsinki.

## Consent for publication

NA

## Availability of data and materials

The datasets supporting the conclusions of this article are available upon request to the corresponding author.

## Abbreviations

ACQ: Juniper asthma control questionnaire
ATS: American Thoracic Society
BD: broncho dilator
BMI: body mass index
FENO: exhaled nitric oxide
FEV_1_: forced expiratory volume in 1 second
FVC: forced vital capacity
GINA: Global Initiative for Asthma Guidelines
ICS: inhaled corticosteroid
MA: moderate asthma
SA: severe asthma
YOA: years of asthma
